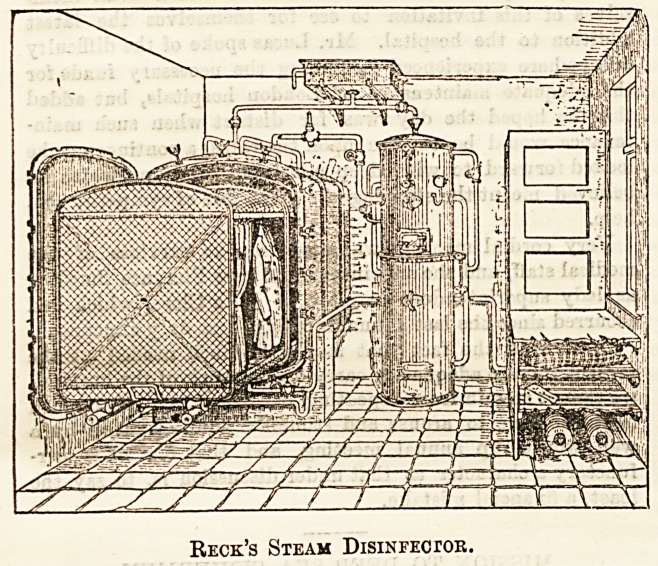# Reck's Steam Disinfector

**Published:** 1895-06-01

**Authors:** 


### The Institutional Workshop.

### PRACTICAL DEPARTMENTS.

### RECK'S STEAM DISINFECTOR.

Inquiries have often reached us from the authorities of cottage hospitals and others interested in the important question of efficient disinfection in country districts as to the best apparatus to procure, and as in such cases funds are as a rule but very limited, and so expensive an arrangement as a Washington Lyon or a Manlove and Alliott high pressure disinfector out of the question on this account, it has been difficult to advise; indeed, in most cases wholesale destruction has seemed the only alternative.

Now we are glad to welcome an apparatus which would appear to be the very thing wanted, and to combine all the most necessary qualifications with a moderation in price which will bring it within the reach of small institutions, for the needs of which it will be found amply sufficient. Captain Reck, of Copenhagen, is the inventor of the apparatus in question, and it is his practical experience in the army which has led to the construction of this useful machine. It is being widely adopted in Denmark and elsewhere,

Reck's Steam Disinfector.

and it is undoubtedly owing to this that disinfection may be said to be carried out more thoroughly in Danish rural districts than in any other country.

Our illustration, which we publish by permission of the Blackman Ventilating Company, 63, Fore Street, E.C., agents in England for Captain Reck's invention, gives a general idea of its appearance. Its special features are (1) the use of low pressure steam, "delivered to the apparatus by an automatic regulator at a rate which cannot be exceeded; (2) the absence of any steam jacket; and (3) a cold shower introduced into the chamber which has for its object the speedy removal of all steam from the interior." This cold shower is prevented from injuring the clothes by a shielding arrangement which distributes the water over a large surface and completely protects the articles from moisture.

A very careful testing of the efficiency of this apparatus was undertaken at the end of last year by Dr. G. Read, medical officer of health, Staffordshire County Council, assisted by other medical men, and the result of their experiments was published in *Public Health* for January of this year. These were eminently satisfactory. The penetrating power of the low pressure steam and the temperatures reached in various thicknesses of material were proved; the amount of moisture left in the articles after the disinfecting process was also tested, and the destructive power of the apparatus upon bacteria, all experiments giving satisfactory results.

The following table shows the temperature reached in various cases:—

| In 15 minutes—Folds of blankets. | | | In 35 minutes. | | |
|---|---|---|---|---|---|
| 4 folds. | 8 folds. | 16 folds. | In chamber. | In 16 folds blankets | Between mattresses. |
| 219° | 218° | 212° | 215—6° | 220° | 211° |

The dryness of material at the completion of the process is proved to be thorough, so that after a shaking out there is practically no moisture retained. With regard to the bacteriological tests, which were conducted by Dr. Barwise, of the Derbyshire County Council, anthrax bacilli and spores, garden soil and human excreta were used, "and with the exception of a few soil bacteria which are highly tenacious of life and survived the process, this part of the experiment was entirely successful, as was proved by control experiments in every case."

By permission of the Blackman Ventilating Company we have inspected one of these disinfectors, and can testify to boiling point being registered by a thermometer placed between several folds of blanket in seven minutes. Thirty-five minutes are recommended as a desirable time for articles to remain in the chamber. The cylinders are made circular, oval, or rectangular in shape, of capacity from 17 to 175 cubic feet; "they are protected against loss of heat by hair felt, they are made of strong steel plates and fitted with two strong doors, one for admission, and the other for delivery. . . . and especially with a . . . reliable steam pressure regulating valve, that automatically keeps an even steam pressure in the disinfectors, quite independently of the boiler pressure."

Dr. Reid points out that a "simple cylinder which has to resist only a small pressure (1½ lb. to the square inch) can be constructed at a much smaller cost than a jacketted high pressure cylinder, and it is this which mainly accounts for the small cost of Reck's apparatus compared with that in use in this country." The cradle which contains the articles to be disinfected is of galvanised wire covered with felt, and it will be observed that the construction being so simple repairs are at all times possible at no great expense.

It is therefore now demonstrated that Captain Reck's disinfector will be of inestimable value in the case of rural districts and small country hospitals, and it is possible to inspect theapparatus in London by applying to the Blackman Ventilating Company, or at Stafford at the depôt of the corporation of Stafford, or at the new hospital at Whitehaven. We should mention that all particulars concerning price, &c., may be obtained upon application. The cost, of course, will vary according as to whether an existing steam boiler can be utilised or not and with regard to size. It is stated that a disinfector with about 50 cubic feet capacity which can be filled anew every 45 or 50 minutes will suffice for a district with a population of from 10,000 to 20,000 inhabitants. The circular form is the cheapest, and well adapted for hospital use, but the oval has in certain respects advantages over it.

To conclude, there is no difficulty experienced in the management of the apparatus, in connection with which every detail has been so well and thoroughly thought out by its inventor that all possibility of accident has been guarded against. Captain Reck's military experiences having evidently shown him that elaborate processes are all very well, but unless it is also possible to have skilled attendance they are of doubtful value in many cases; and it is because of its simplicity that the apparatus in question will be of such immense benefit in places and institutions where a more complicated affair would be unworkable.

## Figures and Tables

**Figure f1:**